# 5-Acetyl-3-hy­droxy-4-phenyl-4,5-dihydro-1*H*-1,5-benzodiazepin-2(3*H*)-one

**DOI:** 10.1107/S1600536811047878

**Published:** 2011-11-16

**Authors:** Mohamed Rida, Abdusalam Alsubari, El Mokhtar Essassi, Hafid Zouihri, Seik Weng Ng

**Affiliations:** a Laboratoire de Chimie Organique Hétérocyclique, Pôle de Compétences Pharmacochimie, Université Mohammed V-Agdal, BP 1014 Avenue Ibn Batout, Rabat, Morocco; b Institute of Nanomaterials and Nanotechnology MAScIR, Avenue de l’Armée Royale, Rabat, Morocco; c Department of Chemistry, University of Malaya, 50603 Kuala Lumpur, Malaysia; d Chemistry Department, King Abdulaziz University, PO Box 80203 Jeddah, Saudi Arabia

## Abstract

In the title compound, C_17_H_16_N_2_O_3_, the seven-membered diazepine ring adopts a boat conformation with the hy­droxy-substituted C atom at the prow and fused benzene ring C atoms at the stern. The phenyl substituent occupies an equatorial position. The amino group of the ring system is a hydrogen-bond donor to the oxo O atom of an inversion-related mol­ecule, and the hy­droxy group is a hydrogen-bond donor to the acetyl O atom of another inversion-related mol­ecule. The two hydrogen bonds generate a ribbon motif parallel to [10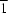] in the crystal structure.

## Related literature

For a related 1,5-benzodiazepin-2(3*H*)-one structure, see: Rida *et al.* (2011 ▶).
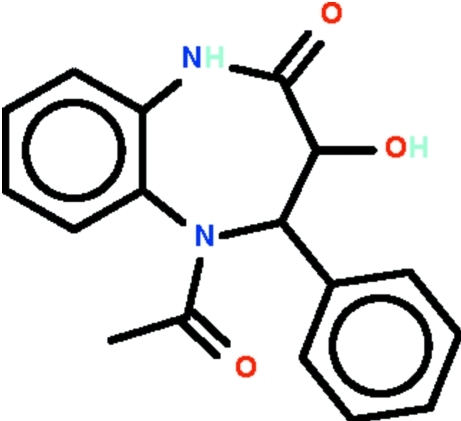


## Experimental

### 

#### Crystal data

C_17_H_16_N_2_O_3_
*M* *_r_* = 296.32
Triclinic, 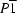

*a* = 8.9710 (1) Å
*b* = 9.3142 (1) Å
*c* = 9.4129 (1) Å
α = 81.563 (1)°
β = 68.921 (1)°
γ = 80.146 (1)°
*V* = 719.95 (1) Å^3^
*Z* = 2
Mo *K*α radiation
μ = 0.10 mm^−1^
*T* = 293 K
0.29 × 0.23 × 0.18 mm

#### Data collection

Bruker APEX DUO diffractometer
19110 measured reflections
4203 independent reflections
3584 reflections with *I* > 2σ(*I*)
*R*_int_ = 0.024

#### Refinement

*R*[*F* ^2^ > 2σ(*F* ^2^)] = 0.047
*wR*(*F* ^2^) = 0.149
*S* = 1.02
4203 reflections
208 parameters
H atoms treated by a mixture of independent and constrained refinement
Δρ_max_ = 0.30 e Å^−3^
Δρ_min_ = −0.27 e Å^−3^

### 

Data collection: *APEX2* (Bruker, 2009 ▶); cell refinement: *SAINT* (Bruker, 2009 ▶); data reduction: *SAINT*; program(s) used to solve structure: *SHELXS97* (Sheldrick, 2008 ▶); program(s) used to refine structure: *SHELXL97* (Sheldrick, 2008 ▶); molecular graphics: *X-SEED* (Barbour, 2001 ▶); software used to prepare material for publication: *publCIF* (Westrip, 2010 ▶).

## Supplementary Material

Crystal structure: contains datablock(s) global, I. DOI: 10.1107/S1600536811047878/lh5376sup1.cif

Structure factors: contains datablock(s) I. DOI: 10.1107/S1600536811047878/lh5376Isup2.hkl

Supplementary material file. DOI: 10.1107/S1600536811047878/lh5376Isup3.cml

Additional supplementary materials:  crystallographic information; 3D view; checkCIF report

## Figures and Tables

**Table 1:** Hydrogen-bond geometry (Å, °)

*D*—H⋯*A*	*D*—H	H⋯*A*	*D*⋯*A*	*D*—H⋯*A*
N1—H1⋯O1^i^	0.89 (2)	2.04 (2)	2.924 (1)	175 (2)
O2—H2⋯O3^ii^	0.83 (2)	2.09 (2)	2.905 (1)	168 (2)

Symmetry codes: (i) 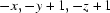; (ii) 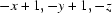.

**Fig. 1.**
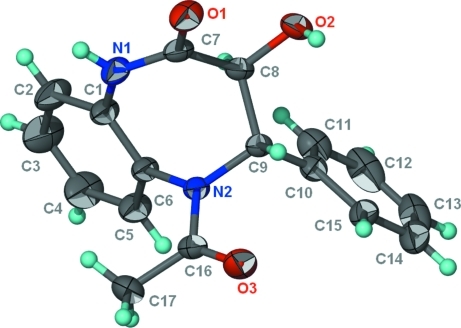
Thermal ellipsoid plot (Barbour, 2001) of C17H16N2O3 at the 50% probability level; hydrogen atoms are drawn as spheres of arbitrary radius.

**Fig. 2.**
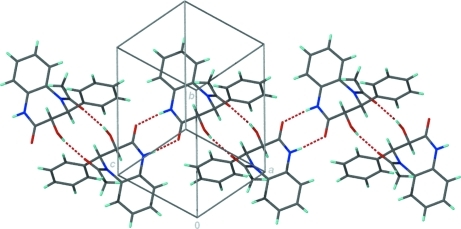
Ribbon motif.

## supplementary crystallographic information

### Comment

The report on 3-hydroxy-4-phenyl-1-[(3-phenyl-4,5-dihydro-1,2-oxazol-5-yl)methyl]-4,5-dihydro-1*H*-1,5-benzodiazepin-2(3*H*)-one provides the preparation and biological activity of this class of benzodiazepin-2-ones (Rida *et al.*, 2011). In the present study, the reactant, 3-ydroxy-4-phenyl-4,5-dihydro-1*H*-1,5-benzodiazepin-2(3*H*)-one, has two amino –NH– units in the ring system; however, only one site is acetylated when the compound is treated with acetic anhydride. In the title compound, the seven-membered diazepine ring adopts a boat conformation with the hydroxy-substituted C atom at the prow and fused-ring C atoms at the stern; the phenyl substituent occupies an equatorial position (Fig. 1). The amino group of the ring system is a hydrogen-bond donor to the oxo O atom of an inversion-related molecule, and the hydroxy group is hydrogen-bond donor to the acetyl O atom of another inversion-related molecule (Table 1). The two hydrogen bonds generate a ribbon motif parallel to [1 0 - 1] (Fig. 2).

### Experimental

3-Hydroxy-4-phenyl-4,5-dihydro-1*H*-1,5-benzodiazepin-2(3*H*)-one (1 g. 3.9 mmol) was heated in acetic anhydride (20 ml) for 12 h. The precipitate was collected and recrystallized from ethanol to afford colorless crystals.

### Refinement

Carbon-bound H-atoms were placed in calculated positions (C–H 0.93–0.98 Å) and were included in the refinement in the riding model approximation, with *U*(H) set to 1.2–1.5*U*(C). The amino and hydroxy H-atoms were located in a difference Fourier map and were freely refined.

### Crystal data

**Table:**

C_17_H_16_N_2_O_3_	*Z* = 2
*M**_r_* = 296.32	*F*(000) = 312
Triclinic, *P*1	*D*_x_ = 1.367 Mg m^−^^3^
Hall symbol: -P 1	Mo *K*α radiation, λ = 0.71073 Å
*a* = 8.9710 (1) Å	Cell parameters from 9950 reflections
*b* = 9.3142 (1) Å	θ = 2.2–34.5°
*c* = 9.4129 (1) Å	µ = 0.10 mm^−^^1^
α = 81.563 (1)°	*T* = 293 K
β = 68.921 (1)°	Prism, colorless
γ = 80.146 (1)°	0.29 × 0.23 × 0.18 mm
*V* = 719.95 (1) Å^3^	

### Data collection

**Table:**

Bruker APEX DUO diffractometer	3584 reflections with *I* > 2σ(*I*)
Radiation source: fine-focus sealed tube	*R*_int_ = 0.024
graphite	θ_max_ = 30.0°, θ_min_ = 2.2°
ω scans	*h* = −12→12
19110 measured reflections	*k* = −13→13
4203 independent reflections	*l* = −13→13

### Refinement

**Table:**

Refinement on *F*^2^	Primary atom site location: structure-invariant direct methods
Least-squares matrix: full	Secondary atom site location: difference Fourier map
*R*[*F*^2^ > 2σ(*F*^2^)] = 0.047	Hydrogen site location: inferred from neighbouring sites
*wR*(*F*^2^) = 0.149	H atoms treated by a mixture of independent and constrained refinement
*S* = 1.02	*w* = 1/[σ^2^(*F*_o_^2^) + (0.0876*P*)^2^ + 0.1414*P*] where *P* = (*F*_o_^2^ + 2*F*_c_^2^)/3
4203 reflections	(Δ/σ)_max_ = 0.001
208 parameters	Δρ_max_ = 0.30 e Å^−^^3^
0 restraints	Δρ_min_ = −0.27 e Å^−^^3^

### Fractional atomic coordinates and isotropic or equivalent isotropic displacement parameters (Å^2^)

**Table:**

	*x*	*y*	*z*	*U*_iso_*/*U*_eq_	
O1	0.18813 (12)	0.41278 (9)	0.39332 (11)	0.0488 (2)	
O2	0.50968 (12)	0.39185 (9)	0.34285 (10)	0.0430 (2)	
O3	0.41433 (13)	0.74792 (11)	−0.06449 (10)	0.0513 (2)	
N1	0.14350 (13)	0.64817 (10)	0.44597 (12)	0.0426 (2)	
N2	0.37829 (11)	0.75893 (9)	0.18338 (9)	0.03178 (19)	
C1	0.19921 (15)	0.78134 (12)	0.44620 (13)	0.0406 (3)	
C2	0.1358 (2)	0.85962 (16)	0.57413 (16)	0.0656 (5)	
H2A	0.0547	0.8253	0.6599	0.079*	
C3	0.1927 (3)	0.98823 (18)	0.5745 (2)	0.0756 (6)	
H3	0.1499	1.0396	0.6609	0.091*	
C4	0.3123 (2)	1.04144 (16)	0.4483 (2)	0.0639 (4)	
H4	0.3501	1.1280	0.4496	0.077*	
C5	0.37548 (17)	0.96508 (13)	0.31996 (15)	0.0468 (3)	
H5	0.4570	0.9999	0.2349	0.056*	
C6	0.31790 (13)	0.83653 (11)	0.31723 (12)	0.0346 (2)	
C7	0.24114 (14)	0.52550 (11)	0.39399 (12)	0.0363 (2)	
C8	0.42230 (13)	0.53221 (11)	0.34285 (11)	0.0329 (2)	
H8	0.4407	0.5843	0.4171	0.039*	
C9	0.48415 (12)	0.61900 (10)	0.18549 (10)	0.0293 (2)	
H9	0.4758	0.5617	0.1099	0.035*	
C10	0.65889 (13)	0.64317 (11)	0.13784 (12)	0.0345 (2)	
C11	0.72509 (17)	0.67606 (14)	0.24003 (18)	0.0493 (3)	
H11	0.6625	0.6825	0.3425	0.059*	
C12	0.8864 (2)	0.69927 (17)	0.1874 (3)	0.0685 (5)	
H12	0.9311	0.7212	0.2555	0.082*	
C13	0.98046 (18)	0.69010 (16)	0.0359 (3)	0.0722 (6)	
H13	1.0876	0.7066	0.0020	0.087*	
C14	0.91519 (18)	0.65653 (16)	−0.0649 (2)	0.0624 (4)	
H14	0.9786	0.6493	−0.1671	0.075*	
C15	0.75549 (15)	0.63361 (13)	−0.01454 (15)	0.0450 (3)	
H15	0.7120	0.6115	−0.0835	0.054*	
C16	0.35154 (14)	0.81314 (12)	0.05077 (12)	0.0371 (2)	
C17	0.24042 (18)	0.95367 (15)	0.05114 (17)	0.0529 (3)	
H17A	0.1983	0.9597	−0.0303	0.079*	
H17B	0.2988	1.0350	0.0367	0.079*	
H17C	0.1532	0.9560	0.1472	0.079*	
H1	0.042 (2)	0.635 (2)	0.498 (2)	0.057 (5)*	
H2	0.517 (2)	0.353 (2)	0.266 (2)	0.065 (5)*	

### Atomic displacement parameters (Å^2^)

**Table:**

	*U*^11^	*U*^22^	*U*^33^	*U*^12^	*U*^13^	*U*^23^
O1	0.0575 (5)	0.0342 (4)	0.0491 (5)	−0.0188 (4)	−0.0046 (4)	−0.0074 (3)
O2	0.0622 (5)	0.0290 (4)	0.0370 (4)	0.0006 (3)	−0.0196 (4)	−0.0017 (3)
O3	0.0650 (6)	0.0559 (6)	0.0302 (4)	0.0024 (4)	−0.0175 (4)	−0.0038 (4)
N1	0.0456 (5)	0.0311 (5)	0.0406 (5)	−0.0124 (4)	0.0015 (4)	−0.0043 (4)
N2	0.0399 (4)	0.0259 (4)	0.0254 (4)	−0.0040 (3)	−0.0067 (3)	−0.0015 (3)
C1	0.0511 (6)	0.0282 (5)	0.0330 (5)	−0.0082 (4)	−0.0009 (4)	−0.0050 (4)
C2	0.0924 (12)	0.0422 (7)	0.0385 (6)	−0.0147 (7)	0.0118 (7)	−0.0120 (5)
C3	0.1156 (15)	0.0465 (8)	0.0507 (8)	−0.0142 (9)	−0.0023 (9)	−0.0248 (6)
C4	0.0897 (11)	0.0368 (6)	0.0631 (9)	−0.0180 (7)	−0.0139 (8)	−0.0185 (6)
C5	0.0571 (7)	0.0316 (5)	0.0465 (6)	−0.0148 (5)	−0.0066 (5)	−0.0058 (4)
C6	0.0432 (5)	0.0258 (4)	0.0298 (5)	−0.0061 (4)	−0.0052 (4)	−0.0040 (3)
C7	0.0491 (6)	0.0288 (5)	0.0270 (4)	−0.0114 (4)	−0.0062 (4)	−0.0004 (3)
C8	0.0469 (5)	0.0254 (4)	0.0259 (4)	−0.0063 (4)	−0.0116 (4)	−0.0009 (3)
C9	0.0385 (5)	0.0249 (4)	0.0240 (4)	−0.0057 (3)	−0.0091 (3)	−0.0028 (3)
C10	0.0389 (5)	0.0254 (4)	0.0381 (5)	−0.0054 (4)	−0.0111 (4)	−0.0034 (4)
C11	0.0556 (7)	0.0410 (6)	0.0608 (8)	−0.0076 (5)	−0.0281 (6)	−0.0112 (5)
C12	0.0610 (9)	0.0441 (7)	0.1198 (16)	−0.0066 (6)	−0.0511 (10)	−0.0159 (8)
C13	0.0406 (7)	0.0385 (7)	0.1285 (17)	−0.0077 (5)	−0.0178 (9)	−0.0063 (8)
C14	0.0456 (7)	0.0411 (7)	0.0778 (10)	−0.0069 (5)	0.0034 (7)	0.0027 (6)
C15	0.0454 (6)	0.0376 (6)	0.0426 (6)	−0.0069 (5)	−0.0042 (5)	−0.0013 (4)
C16	0.0414 (5)	0.0350 (5)	0.0313 (5)	−0.0071 (4)	−0.0097 (4)	0.0036 (4)
C17	0.0566 (7)	0.0426 (6)	0.0510 (7)	0.0028 (5)	−0.0171 (6)	0.0076 (5)

### Geometric parameters (Å, °)

**Table:**

O1—C7	1.2261 (13)	C7—C8	1.5297 (16)
O2—C8	1.4046 (13)	C8—C9	1.5382 (13)
O2—H2	0.83 (2)	C8—H8	0.9800
O3—C16	1.2247 (14)	C9—C10	1.5148 (14)
N1—C7	1.3510 (15)	C9—H9	0.9800
N1—C1	1.4149 (14)	C10—C11	1.3897 (17)
N1—H1	0.89 (2)	C10—C15	1.3905 (16)
N2—C16	1.3637 (14)	C11—C12	1.395 (2)
N2—C6	1.4302 (13)	C11—H11	0.9300
N2—C9	1.4776 (12)	C12—C13	1.378 (3)
C1—C2	1.3886 (17)	C12—H12	0.9300
C1—C6	1.3950 (15)	C13—C14	1.375 (3)
C2—C3	1.381 (2)	C13—H13	0.9300
C2—H2A	0.9300	C14—C15	1.3818 (19)
C3—C4	1.378 (3)	C14—H14	0.9300
C3—H3	0.9300	C15—H15	0.9300
C4—C5	1.3814 (19)	C16—C17	1.5031 (17)
C4—H4	0.9300	C17—H17A	0.9600
C5—C6	1.3897 (15)	C17—H17B	0.9600
C5—H5	0.9300	C17—H17C	0.9600
			
C8—O2—H2	109.8 (14)	N2—C9—C10	111.43 (8)
C7—N1—C1	124.00 (10)	N2—C9—C8	109.66 (8)
C7—N1—H1	114.4 (12)	C10—C9—C8	113.46 (9)
C1—N1—H1	120.4 (12)	N2—C9—H9	107.3
C16—N2—C6	122.88 (9)	C10—C9—H9	107.3
C16—N2—C9	118.55 (8)	C8—C9—H9	107.3
C6—N2—C9	118.41 (8)	C11—C10—C15	119.09 (12)
C2—C1—C6	119.01 (11)	C11—C10—C9	122.52 (10)
C2—C1—N1	120.58 (11)	C15—C10—C9	118.38 (10)
C6—C1—N1	120.40 (10)	C10—C11—C12	119.33 (15)
C3—C2—C1	120.25 (13)	C10—C11—H11	120.3
C3—C2—H2A	119.9	C12—C11—H11	120.3
C1—C2—H2A	119.9	C13—C12—C11	120.92 (16)
C4—C3—C2	120.84 (13)	C13—C12—H12	119.5
C4—C3—H3	119.6	C11—C12—H12	119.5
C2—C3—H3	119.6	C14—C13—C12	119.71 (14)
C3—C4—C5	119.44 (13)	C14—C13—H13	120.1
C3—C4—H4	120.3	C12—C13—H13	120.1
C5—C4—H4	120.3	C13—C14—C15	120.03 (16)
C4—C5—C6	120.37 (12)	C13—C14—H14	120.0
C4—C5—H5	119.8	C15—C14—H14	120.0
C6—C5—H5	119.8	C14—C15—C10	120.91 (14)
C5—C6—C1	120.05 (10)	C14—C15—H15	119.5
C5—C6—N2	121.01 (10)	C10—C15—H15	119.5
C1—C6—N2	118.94 (9)	O3—C16—N2	120.90 (10)
O1—C7—N1	122.08 (11)	O3—C16—C17	121.05 (11)
O1—C7—C8	121.49 (10)	N2—C16—C17	118.04 (10)
N1—C7—C8	116.36 (9)	C16—C17—H17A	109.5
O2—C8—C7	111.68 (8)	C16—C17—H17B	109.5
O2—C8—C9	110.91 (8)	H17A—C17—H17B	109.5
C7—C8—C9	111.53 (9)	C16—C17—H17C	109.5
O2—C8—H8	107.5	H17A—C17—H17C	109.5
C7—C8—H8	107.5	H17B—C17—H17C	109.5
C9—C8—H8	107.5		
			
C7—N1—C1—C2	−134.65 (15)	C6—N2—C9—C10	−87.10 (10)
C7—N1—C1—C6	45.87 (19)	C16—N2—C9—C8	−145.05 (10)
C6—C1—C2—C3	−1.6 (3)	C6—N2—C9—C8	39.37 (12)
N1—C1—C2—C3	178.87 (17)	O2—C8—C9—N2	173.40 (8)
C1—C2—C3—C4	0.3 (3)	C7—C8—C9—N2	48.24 (11)
C2—C3—C4—C5	0.1 (3)	O2—C8—C9—C10	−61.29 (11)
C3—C4—C5—C6	0.7 (3)	C7—C8—C9—C10	173.56 (8)
C4—C5—C6—C1	−2.0 (2)	N2—C9—C10—C11	84.57 (12)
C4—C5—C6—N2	177.86 (14)	C8—C9—C10—C11	−39.79 (14)
C2—C1—C6—C5	2.5 (2)	N2—C9—C10—C15	−94.64 (11)
N1—C1—C6—C5	−178.04 (12)	C8—C9—C10—C15	140.99 (10)
C2—C1—C6—N2	−177.42 (13)	C15—C10—C11—C12	0.26 (18)
N1—C1—C6—N2	2.06 (18)	C9—C10—C11—C12	−178.95 (11)
C16—N2—C6—C5	−67.48 (16)	C10—C11—C12—C13	0.0 (2)
C9—N2—C6—C5	107.89 (12)	C11—C12—C13—C14	−0.5 (2)
C16—N2—C6—C1	112.42 (13)	C12—C13—C14—C15	0.7 (2)
C9—N2—C6—C1	−72.21 (14)	C13—C14—C15—C10	−0.4 (2)
C1—N1—C7—O1	−178.75 (11)	C11—C10—C15—C14	−0.10 (18)
C1—N1—C7—C8	4.08 (17)	C9—C10—C15—C14	179.14 (11)
O1—C7—C8—O2	−18.12 (14)	C6—N2—C16—O3	175.01 (10)
N1—C7—C8—O2	159.07 (10)	C9—N2—C16—O3	−0.35 (16)
O1—C7—C8—C9	106.61 (11)	C6—N2—C16—C17	−6.10 (16)
N1—C7—C8—C9	−76.20 (12)	C9—N2—C16—C17	178.53 (10)
C16—N2—C9—C10	88.48 (11)		

### Hydrogen-bond geometry (Å, °)

**Table:**

*D*—H···*A*	*D*—H	H···*A*	*D*···*A*	*D*—H···*A*
N1—H1···O1^i^	0.89 (2)	2.04 (2)	2.924 (1)	175 (2)
O2—H2···O3^ii^	0.83 (2)	2.09 (2)	2.905 (1)	168 (2)

Symmetry codes: (i) −*x*, −*y*+1, −*z*+1; (ii) −*x*+1, −*y*+1, −*z*.
